# Prevalence of *Salmonella* contamination in consumed eggs in Iran: A systematic review and meta-analysis study on published studies from 1996 to 2018

**DOI:** 10.14202/vetworld.2020.2743-2751

**Published:** 2020-12-22

**Authors:** Behnam Hosseininezhad, Enayat Berizi, Marzieh Nader, Seyed Mohammad Mazloomi, Saeid Hosseinzadeh, Laya Ebrahimi, Morteza Zare

**Affiliations:** 1 Nutrition Research Center, Department of Food Hygiene and Quality Control, School of Nutrition and Food Sciences, Shiraz University of Medical Sciences, Shiraz, Iran; 2 Department of Food Hygiene and Public Health, School of Veterinary Medicine, Shiraz University, Shiraz, Iran

**Keywords:** eggs, Iran, *Salmonella*, systematic review and meta-analysis

## Abstract

**Background and Aim:**

Food poisoning caused by *Salmonella* is among the most common gastrointestinal discomfort resulted from egg consumption which can produce various syndromes. The present study is a systematic review and meta-analysis investigation on the published studies about the prevalence of *Salmonella* contamination in the consumed eggs in Iran.

**Materials and Methods:**

The data were collected and analyzed from four international search databases, including PubMed, Scopus, Science Direct, and Google Scholar and four Iranian databases comprising SID, MagIran, Civilica, and IranDoc. After searching all the databases, 303 articles were found, from which 31 articles were included in the final analysis.

**Results:**

According to the data analysis, the highest rate of contamination was belonged to the industrial eggs (7.49%), however, the prevalence rate was reported 13.61% in the eggshell part. The overall prevalence of *Salmonella* contamination in consumed eggs of Iran using culture of microbial, molecular, molecular-serological, culture-molecular, culture-serological, and culture -molecular-serological methods was obtained 11.33%, 5.52%, 0.37%, 1.91%, 5.52%, and 0.73%, respectively. Prevalence in the 21 geographical areas, where studies have been conducted, ranged from 0% (Zahedan) to 29.06% (Tabriz). The studies have also showed that eight different serotypes were among the major cause of *Salmonella* contamination in eggs. The most common *Salmonella* serotype was *Salmonella* Enteritidis and the highest diversity in *Salmonella* contaminant serotypes was recorded in Talesh (including *S*. Enteritidis, *Salmonella* Gallinarum, *Salmonella* Vircho*w*, and *Salmonella* Newport).

**Conclusion:**

Results of this study revealed the high prevalence of *Salmonella* contamination in eggs, in Iran. Therefore, disinfection and cleaning bed, cleaning of equipment and supplies, and proper maintenance temperature and humidity of the eggs are recommended. In addition, proper personal hygiene and prohibition of consuming raw egg products are essential.

## Introduction

*Salmonella* is the most common and important bacteria involved in food poisoning caused by egg consumption and can produce various syndromes, with the most common clinical manifestations of gastroenteritis and food poisoning [1]. Salmonellosis in humans and animals is generated by various serotypes through oral intake [1,2]. *Salmonella* Enteritidis and *Salmonella* Typhimurium are the most common serotypes involved in the occurrence of salmonellosis [3] through consumption of poultry meat and egg [4]. In general, there are two different routes to transfer *Salmonella* to the egg. The initial transmission rout is vertical which is happening through the direct contamination of the yolk, whites, membrane, or shell of the egg before ovulation. Horizontal transmission is the second rout, in which, the bacterium contaminates the shell surface through penetrating the infected intestine or stool [5]. Of two common serotypes, contamination with *S*. Enteritidis and *S*. Typhimurium was occurred during ovulation [3]. Unfortunately, massive use of antibiotics in human and in the livestock and poultry breeding industries has led to a dramatic increase in *Salmonella* resistance strains, which is a global dilemma [2]. This issue has doubled the importance of attention to the health of red meat, poultry meat, and eggs. The prevalence of egg contamination with *Salmonella* has been investigated in different studies in Iran which was varied from 0% to 99% [2,6]. Amin-Zare *et al*. [7] investigated the contamination to *Salmonella* in 100 samples of industrial eggs in Urmia by microbial culture method. The results showed that the contents of six samples were contaminated with *S*. Enteritidis. In addition, no evidence of *Salmonella* contamination in 525 industrial egg specimens was reported in Isfahan [8].

Understanding the importance of prevalence, the involved serotypes of *Salmonella* and also contamination of different parts of the egg to promote preventive measures of salmonellosis is crucial.

Therefore, the purpose of this systematic review and meta-analysis study was to access the above-mentioned information through a survey of accomplished articles and studies in Iran.

## Materials and Methods

### Ethical approval

This is a systematic review and meta-analysis of the published studies and ethical approval is not needed for this study.

### Study method

The present study was a systematic review and meta-analysis on the prevalence of *Salmonella* contamination of the eggs consumed in Iran from 1996 to 2018.

### Search strategy

In 2018, a comprehensive scientific search was carried out in four valid international databases (PubMed, Scopus, Science Direct, and Google Scholar) and four Iranian valid electronic databases (Sid, MagIran, Civilica, and IranDoc). The selected keywords for the international and national databases were “*Salmonella*,” “egg,” and “Iran.” The collected data were then entered into the EndNote, X8 software, to automatically delete the duplicate articles.

### Screening

Initial search of studies was conducted by two authors (first and last ones). Screening of studies, extraction of results, and evaluation of the quality control of articles were performed separately by two authors (first and last ones). If there was no agreement between the two authors, the team leader (the responsible author) would declare a final comment on that article.

### Inclusion and exclusion criteria

Among the extracted studies, some of them were excluded from the list of received studies: Review articles, summary of presented articles at the congress, the studies that abstract and full texts were not available, surveys that were not part of the original research, unrelated surveys to the prevalence of *Salmonella* contamination in consumed eggs, and studies that did not explicitly express data.

### Quality control

A checklist developed by the Joanna Briggs Institute was used to check and control the quality of articles [9]. This tool consists of eight questions that are classified as Yes, No, Uncertain, and Unused. The purpose of this tool is to evaluate the methodological quality of studies, ways to access and understand the errors available in studies, design, implementation, and analysis of data.

### Data extraction

From each article, some information including authors’ name, type of egg (local or industrial), method of detection, relevant *Salmonella* serotypes, egg contamination site, frequency rate of positive cases, as well as areas contaminated with *Salmonella* were entered into the pre-designed tables (Table-1) [1-8,10-32]. Then, the data were classified and statistically analyzed.

**Table-1:** Information of included studies in the meta-analysis of the prevalence of *Salmonella* spp. in eggs in Iran.

Egg type	N a	p (%)	95% confidence interval	Egg Cs	Method	Location	Serotype	References
Industrial	120	3.3	0.2-6.4	Contents	Culture	Mashhad	Spp.	[1]
Local	120	7.5	2.9-12.1	Contents	Culture	Mashhad	Spp.	
Industrial	120	22.5	15.2-29.8	Shell	Culture	Mashhad	Spp.	
Local	120	39.1	30.6-47.6	Shell	Culture	Mashhad	Spp.	
Industrial	100	5	1.1-8.9	Shell	Culture	Qom	Spp.	[15]
Industrial	100	1	0-2.8	Contents	Culture	Qom	Spp.	
Industrial	100	0	0-4.5	Contents	Culture	Talesh	Spp.	[2]
Local	100	0	0-4.5	Contents	Culture	Talesh	Spp.	
Industrial	100	19	11.5-26.5	Shell	Culture	Talesh	*Enteritidis*	
Local	100	4	0.7-7.3	Shell	Culture	Talesh	Spp.	
Local	210	3.3	1.4-5.2	Shell	PCR	Kohgiluyeh and Boyer-Ahmad	Spp.	[13]
Local	210	3.3	1.4-5.2	Contents	PCR	Kohgiluyeh and Boyer-Ahmad	Spp.	
Industrial	150	1.3	0-2.8	Contents/shell	Culture/PCR	Shahrud	Spp.	[16]
Local	150	2.5	0.7-4.5	Contents/shell	Culture/PCR	Shahrud	Spp.	
Industrial	186	1.6	0.1-3.1	Contents	Culture/PCR	Esfahan	*Enteritidis*	[17]
Local	500	0.4	0.1-0.7	Shell	Culture/PCR/Serology	Birjand	Spp.	[4]
Local	500	0.2	0-0.5	Contents/shell	Culture/PCR/serology	Birjand	Spp.	
Industrial	100	6	1.5-10.5	Contents	Culture	Urmia	*Enteritidis*	[7]
Industrial	120	56.6	47.9-65.1	Shell	Culture/serology	Zanjan	Spp.	[18]
Industrial	120	0	0-2.9	Contents	Culture/serology	Zanjan	Spp.	
Local	54	1.8	0-5.3	Contents	PCR	Mazandaran	*Enteritidis*	[19]
Local	54	1.8	0-5.3	Shell	PCR	Mazandaran	*Enteritidis*	
Industrial	625	4		Contents	Culture/PCR/serology	Tehran	*Enteritidis*	[11]
Industrial	1680	14.2	12.6-15.8	Contents	Culture	Gilan Zanjan…	Spp.	[20]
Industrial	100	2	0.1-3.9	Contents	Culture/serology	Tehran	*Enteritidis*	[21]
Industrial	100	8	Mar-13	Shell	Culture/serology	Tehran	*Enteritidis*	
Industrial	120	15.8	9.4-22.2	Contents	Culture	Tabriz	Spp.	[6]
Industrial	120	99.1	97.5-100	Shell	Culture	Tabriz	Spp.	
Industrial	40	22.5	16.2-28.8	Contents	PCR	Karaj	*Enteritidis*	[10]
Local	100	5	1.1-8.9	Contents	Culture	Ahvaz	Spp.	[22]
Local	100	4	0.7-7.3	Shell	Culture	Ahvaz	Spp.	
Industrial	180	0	0-2.4	Shell	Culture/PCR/serology	Khorramabad	Spp.	[5]
Local	180	0	0-2.4	Contents	Culture/PCR/serology	Khorramabad	Spp.	
Local	180	1.1	0-2.6	Contents	Culture/PCR/serology	Khorramabad	Spp.	
Industrial	180	0	0-2.4	Shell	Culture/PCR/serology	Khorramabad	Spp.	
Industrial	775	0.6	0.1-1.1	Shell	Culture	Ahvaz	Spp.	[23]
Industrial	775	0.1	0-0.3	Contents	Culture	Ahvaz	Spp.	
Industrial	775	0.1	0-0.3	Contents/shell	Culture	Ahvaz	Spp.	
Industrial	230	0	0-1.9	Contents	Culture	Zahedan	Spp.	[24]
Industrial	100	20	12.2-27.8	Shell	Culture	Urmia	Spp.	[25]
Local	100	50	40.2-59.8	Shell	Culture	Urmia	Spp.	
Industrial	100	2	0.1-3.9	Contents	Culture	Urmia	Spp.	
Local	100	29	20.3-37.7	Contents	Culture	Urmia	Spp.	
Local	300	0.3	0-0.9	Shell	Culture/serology	Gilan	Spp.	[14]
Local	300	1	0-2.1	Contents	Culture/serology	Gilan	Spp.	
Local	60	1.6	0-4.3	Contents	Culture/serology	Qom	Spp.	[12]
Industrial	60	0	0-7.02	Contents	Culture/serology	Qom	Spp.	
Industrial	34	0	0-12.2	Contents	Culture/PCR	Tehran	Spp.	[26]
Local	200	5	2.3-7.7	Contents	Culture/PCR	Fasa	*Enteritidis*	[27]
Industrial	500	0.4	0-0.7	Shell	Culture	Shiraz	Spp.	[28]
Industrial	500	0.2	0-0.5	Contents	Culture	Shiraz	Spp.	
Local	120	1.6	0-3.8	Contents	Culture/serology	Zanjan	Spp.	[29]
Industrial	120	0	0-3.7	Shell	Culture/serology	Zanjan	Spp.	
Local	120	0	0-3.7	Shell	Culture/serology	Zanjan	Spp.	
Industrial	120	0	0-3.7	Contents	Culture/serology	Zanjan	Spp.	
Local	50	10	5.8-14.2	Contents	Culture/PCR	Shiraz	*Enteritidis*	[30]
Industrial	150	1.3	0-3.1	Shell	Culture/PCR	Tabriz	Spp.	[31]
Industrial	150	0	0-2.9	Contents	Culture/PCR	Tabriz	Spp.	
Industrial	100	0	0-4.4	Contents	Culture	Shahrekord	Spp.	[32]
Industrial	250	1.6	0.1-3.1	Shell	Culture/PCR	Mashhad	Spp.	[3]
Industrial	250	0	0-1.7	Contents	Culture/PCR	Mashhad	Spp.	
Industrial	525	0	0-0.7	Contents	Culture	Isfahan	Spp.	[8]
Industrial	525	0	0-0.7	Shell	Culture	Isfahan	Spp.	

a =Number of samples,

b= Number of positive samples, PCR=Polymerase chain reaction

### Risk of bias between studies

Egger’s test was used to investigate the risk of propagation bias [33].

### Statistical analysis

Chi-square test with a significance level of 0.05, I^2^ >50% was used to assess the degree of heterogeneity among the included studies. If there was heterogeneity, the random effect model was used with the inverse variance method. If not, the fixed effect model was applied. All analyses were performed using the statistical software STATA, version 13 (StataCorp LLC, College Station, Texas, USA).

## Results

### Systematic review results

#### Search results and selection of studies

**Figure-1:**
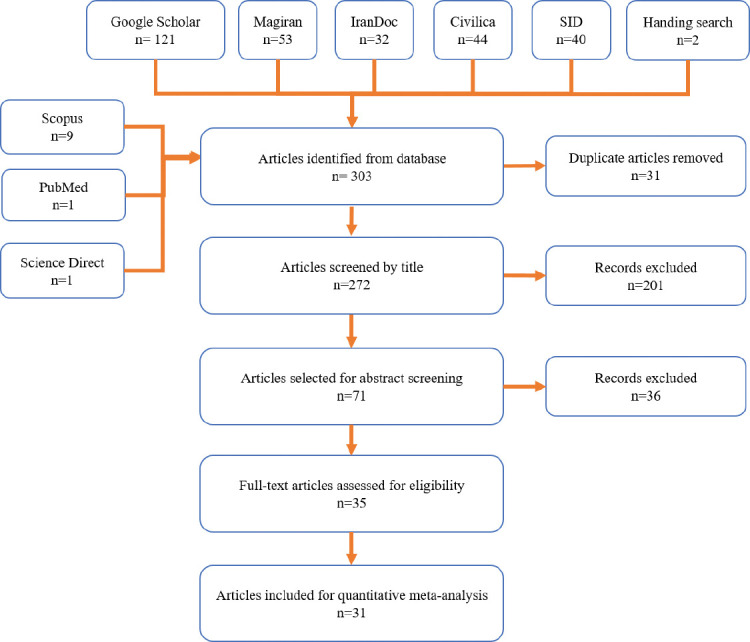
Flowchart of the included eligible studies in systematic review and meta-analysis.

After investigating the international and internal databases, 303 relevant articles were chosen, 272 articles were undergone an assessment of titles and abstracts, after excluding the duplicate articles. After assessment titles and abstracts, 35 articles entered the next stage, in which, the full text of the articles was investigated and 31 articles were approved and entered into the final analysis. During screening stages, some studies were excluded from investigation due to the unrelated subject, the study population, and duplicate results. The flowchart of the included studies is shown in Figure-1.

### Characteristics of studies and data extraction

In this study, the contamination rates were investigated in 21 geographical regions of Iran. However, most studies were accomplished in Zanjan and Mashhad (six studies) (Table-2). Of the studies, 29, 9, and 3 cases were, respectively, examined the contamination of the contents, shell, and the contents and eggshell with *Salmonella* (Table-1). Six different methods, including the culture of microbial, molecular, molecular culture, serological-molecular, culture-serological-molecular, and culture-serological have been used to detect the microorganism (Table-3). The highest prevalence was ranged from 99.1% and 0% in seven studies conducted on egg contamination with *Salmonella* (Table-1).

**Table-2:** Prevalence of *Salmonella* subgrouped by location.

Location	Total inputs	Total sample size	Overall prevalence (%)	95% confidence interval	I^2^ (%)	p for χ^2^
Tehran	4	859	3.50	1.39-5.60	57.3	0.071
Tabriz	4	540	29.06	30.94-89.06	100	0.000
Shiraz	3	1050	0.73	0.20-1.66	90.6	0. 000
Esfahan	3	1236	0.14	0.33-0.61	52.6	0.121
Urmia	5	500	20.78	7.22-34.35	97.0	0.000
Shahrekord	1	100	0.00	−2.50 2.5	0	0
Talesh	4	400	4.45	−0.51 9.42	88.3	0.000
Mashhad	6	980	10.24	5.14-15.35	95.9	0.000
Qom	4	320	1.60	−0.02-3.21	24.3	0.266
Kohgiluyeh and Boyer-Ahmad	2	420	3.30	1.96-4.64	0.0	1.000
Birjand	2	1000	0.30	0.09-0.51	0.0	0.356
Zanjan	6	720	7.35	1.84-12.85	97.0	0.000
Shahrood	2	300	1.81	0.57-3.06	9.7	0.293
Mazandaran	2	108	1.80	−0.67-4.27	0.0	1.000
Gilan	2	600	0.49	−0.12-1.10	16.6	0.274
Karaj	1	40	22.50	16.20-28.80	0	0
Khorramabad	4	720	0.25	−0.46-0.96	0.0	0.659
Ahvaz	4	1750	0.32	−0.06-0.69	72.9	0.005
Zahedan	1	230	0.00	−1.10-1.10	0	0
Fasa	1	200	5.00	2.30-7.70	0	0
Gilan, Zanjan, Kermanshah, Azerbaijan-Shargi, Mazandaran	1	1680	14.20	12.60-15.80	0	0

**Table-3:** Prevalence of *Salmonella* in eggs subgrouped by method of the detection of *Salmonella*.

Method of detection	Total inputs	Total sample size	Overall prevalence (%)	95% confidence interval	I^2^ (%)	p for χ^2^
Culture	29	8405	11.33	7.88-14.78	93.9	0.000
PCR	4	568	5.52	1.63-9.42	89.1	0.000
Culture/PCR	10	1570	1.91	0.75-3.07	74.0	0.000
Culture/PCR/serology	7	2345	0.73	0.08-1.38	81.8	0.000
Culture/serology	8	960	5.52	2.36-8.69	96.0	0.000
PCR/serology	4	480	0.37	−0.69-1.43	0.0	0.669

PCR=Polymerase chain reaction

In the accomplished study, local and industrial eggs have been considered, which the highest prevalence has been reported for industrial eggs. Local eggs are produced by fed on organic diet without any supplementation of chemicals and antibiotic with free access to the outdoors, and industrial eggs produced by hens confined indoors and living in group cages fed on a formulated diet with some supplementation. *Salmonella* distinct serotypes from eggs including *Salmonella* Enteritidis, *Salmonella* Gallinarum, *Salmonella* Virchow, *Salmonella* Newport, *Salmonella* Typhi A, *Salmonella* Agona, *Salmonella* Paratyphi, and *Salmonella* Typhimurium (Table-1).

## Meta-analysis results

### Overall prevalence

**Figure-2:**
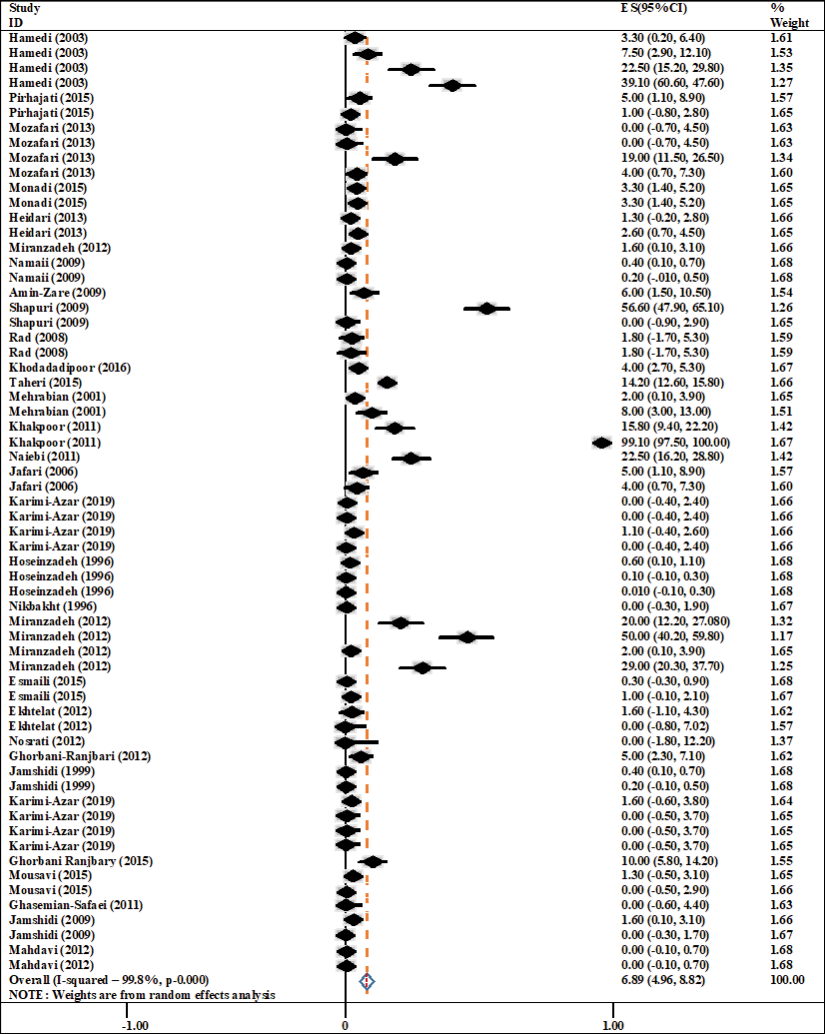
Forest plot of total prevalence outbreak of *Salmonella* in eggs.

The results of meta-analysis showed that the overall prevalence of *Salmonella* in eggs was 6.89% (CI: 4.96%-8.82%) (Figure-2).

### *Salmonella* serotypes

The results showed that the contamination of eggs with *Salmonella* spp. (6.87%) was higher than *S*. Enteritidis (6.20%) (Table-4).

**Table-4:** Prevalence of *salmonella* in eggs subgrouped by type of *Salmonella*.

Serotypes	Total inputs	Total sample size	Overall prevalence (%)	95% confidence interval	I^2^ (%)	p for χ^2^
*Salmonella*. Spp.	52	12,606	6.87	4.74-9.00	99.8	0.000
*Salmonella* Enteritidis	11	1609	6.20	3.84-8.55	87.0	0.000

### Egg contamination site

The meta-analysis revealed that the highest and lowest contaminations were belonged to part of the eggshells (13.61%) and eggshell plus egg contents (0.35%) (Table-5).

**Table-5:** Prevalence of *Salmonella* in eggs subgrouped by egg contamination site of *Salmonella*.

Egg contamination site	Total inputs	Total sample size	Overall prevalence (%)	95% confidence interval	I^2^ (%)	p for χ^2^
Contents	35	7909	2.53	1.77-3.29	93.5	0.000
Shell	24	5044	13.61	7.85-19.37	99.9	0.000
Shell/contents	4	1575	0.35	−0.09-0.78	66.5	0.030

### Method of detection

Among the six methods of detection, the highest prevalence rate of eggs has been confirmed by the conventional microbial culture (11.33%) and the lowest prevalence rate of eggs was observed in combined serological-molecular method (0.37%) (Table-3).

### Geographical location

The highest prevalence rate has been reported in north and northwestern cities (29.06%), Karaj (22.50%), and Urmia (20.78%), moreover, the prevalence rate of 0% has been reported from Sistan-Baluchistan province (Table-2).

## Discussion

The present study showed a relatively high rate of overall prevalence of *Salmonella* (6.89%) in the industrial Iranian eggs (Figure-2). The contamination rate in the eggshell was higher than the contents and the lowest contamination rate was associated with the whole eggs (Table-5). The obtained results showed that in the collected studies, six different methods have been used to detect *Salmonella* with different findings so that the highest and lowest prevalence were, respectively, reported in the microbial culture and the combined serological-PCR methods (Table-3). Eight different serotypes of *Salmonella* were involved in egg contamination with the highest prevalence of *S*. Enteritidis (Table-4). In addition, the highest diversity of *Salmonella* contaminant serotypes (including *S*. Enteritidis, *S*. Gallinarum, *S*. Virchow, and *S*. Newport) was reported from Talesh [2]. Among industrial and local eggs, the highest prevalence was reported in the industrial ones. However, no significant association was observed between prevalence rate and geographic areas (Table-2).

Eggs are produced both locally and industrially, and bacterial contamination occurs in both local and industrially produced eggs. In the present study, the highest prevalence rate was reported for industrial eggs (7.49%) (Table-6). Some of the important reasons are including the misuse of antibiotics (which make *Salmonella* resistant to unfavorable environmental conditions) in poultry farms, high density at industrial poultry breeding location, feeding methods, and different slaughter methods [2].

**Table-6:** Prevalence of *Salmonella* subgrouped by egg type.

Egg type	Total inputs	Total sample size	Total sample size	95% confidence interval	I^2^ (%)	p for χ^2^
Industrial	39	10,500	10,500	4.65-10.33	99.8	0
Local	24	4028	4028	2.22-4.11	92.4	0

Each egg contains two parts: The shell and contents, which both parts can be contaminated to an important pathogen such as *Salmonella* [1]. In the present study, the highest prevalence rate of *Salmonella* was reported in eggshells (13.61%) (Table-5). Naturally, neglecting the health of poultry farm staff (direct contact of hand with the eggshell), inappropriate substrates, contact of chicken feces with the eggshell, and improper maintenance conditions of temperature can lead to grow *Salmonella* on the egg surface [1,5]. In the study of Suresh *et al*. [34] of the 492 eggs studied in South India, 38 cases were positive, with the highest prevalence of the eggshells (29 cases). This finding was consistent with the present study. In another study, more than 5700 eggs from 15 flock contaminated with *S*. Enteritidis were tested, in which the contents of 32 eggs (0.6%) were contaminated. It was also shown the lower prevalence rate in the contents of the eggs than our studies. Different prevalence reported before was probably related to the maintenance conditions of chicken and eggs and overall sanitation [7].

The most important bacterial species involved in eggs are different *Salmonella* serotypes. In this study, *Salmonella* serotypes were divided into two main groups, including *S*. Enteritidis and other *Salmonella* serotypes. *S*. Enteritidis was the dominant serotype involved in the contamination of eggs (Table-1). In various accomplished studies in the world, the most common *Salmonella* serotype separated from contaminated eggs is *S*. Enteritidis [4], which may be due to the important role of flagella in this bacterium to transmit and survive in the host cells [10].

To identify *Salmonella* serotypes, different methods have been used. In this study, six different methods were used to identify different *Salmonella* serotypes. Microbial culture was the most common method and combined culture-serological-PCR assay was the least methods to identify *Salmonella*. The prevalence rate of *Salmonella* in each method was reported differently; for instance the highest prevalence rate was related to microbial culture (11.33%) and the lowest prevalence was associated with the combined serological-PCR method (0.37%) (Table-3). Using microbial culture is common in laboratories due to its easy access and low cost method [35], even though the disadvantage of such techniques is the impairment to detect small numbers of bacteria, time-consuming, and lack of sufficient diagnostic specificity [36]. Since the molecular method has multiple applications [11], it is highly accurate and sensitive and is considered fast and less expensive method, it is also one of the new approaches to confirm the conventional techniques [2,37].

To identify the pathogenic aerobic bacteria from egg shell and its contents, contamination to *Salmonella* serotypes among 6.67% of eggshell was detected using microbial culture method, while none of the eggs contents were found positive. However, seven samples of egg contents and 14 samples of eggshells were reported positive, using PCR technique, the difference was corresponds to the employment of different methods of identification [5]. The difference was due to high sensitivity and accuracy of the molecular methods [37]. The overall prevalence of *Salmonella* in the Iranian eggs was 6.89%. The low prevalence was probably due to the conventional culture used in our study. The results obtained from this study also approve this hypothesis that the prevalence of *Salmonella* was higher if more accurate methods such as combined conventional culture and PCR procedures were employed. Moreover, no significant difference was observed between various geographical areas in Iran. The highest and lowest prevalence were, respectively, reported from Tabriz (29.06%) and Zahedan (0%) (Table-2). The difference was likely due to the inappropriate temperature and humidity [4].

This is the first study that investigates the overall prevalence of *Salmonella* in Iranian eggs. The contamination rate of different parts of egg, common sites of contamination, and the most common serotypes involved are among the most important factors involving in egg contamination. In this study, both local and industrial eggs were examined. In addition, the contamination was reported from many geographical areas of the country. All feasible methods for the diagnosis of *Salmonella* were analyzed and the prevalence of *Salmonella* was also determined in each method. The contamination with all serotypes of the bacteria was identified. The present study was composed of four English and four Persian databases.

In this study, the contamination of shell and contents in the industrial and local eggs was not investigated separately. Few recently published data and Ph.D. thesis are also not included in our analysis.

## Conclusion and Recommendation

The present study was performed based on a systematic review and meta-analysis to evaluate the overall prevalence of *Salmonella* in Iranian eggs. The obtained results showed a relatively high prevalence rate. *S*. Enteritidis was also the most common contaminant serotype. The prevalence of *Salmonella* in the industrial eggs was higher than the local eggs and it was higher in the eggshells. The contamination was observed in most provinces. The results also revealed that six different methods were commonly used to diagnose the bacteria. In most studies, the conventional culture was used which showed higher contamination rate compared, whereas the lowest prevalence was obtained in the molecular-serological method.

To prevent the occurrence of *Salmonella* in the eggs, the following schemes need to be carefully investigated; personal hygiene, disinfection and cleaning bed (in traditional breeding) and cages (in industrial breeding), disinfection and cleaning of equipment and supplies which is in contact with eggs, and observance of proper maintenance conditions (temperature and humidity) [12]. The misuse of antibiotics in the poultry farms should be avoided [13] and also consuming raw and medium (half-cooked) eggs should be prohibited, and if in case, the raw eggs are used in sauces or desserts, lowering pH of the product is strongly recommended [14].

## Authors’ Contributions

EB: Study design; review relevant articles, analysis, and interpretation of data; drafting and finalizing the manuscript; and study supervision. BH, MN, and SH: Review relevant articles, analysis, and interpretation of data; drafting the manuscript. SMM, SH, LE, and MZ: Analysis and interpretation of data; drafting the manuscript. All authors read and approved the final manuscript.
